# Beta, but Not Gamma, Band Oscillations Index Visual Form-Motion Integration

**DOI:** 10.1371/journal.pone.0095541

**Published:** 2014-04-29

**Authors:** Charles Aissani, Jacques Martinerie, Lydia Yahia-Cherif, Anne-Lise Paradis, Jean Lorenceau

**Affiliations:** 1 Université Pierre et Marie Curie, Centre de Recherche de l'Institut du Cerveau et de la Moelle épinière, CNRS UMR7225, Paris, France; 2 CENIR, Centre de Recherche de l’Institut du Cerveau et de la Moelle épinière, Université Pierre et Marie Curie-Paris6, INSERM U975, CNRS UMR7225, Paris, France; University of Tuebingen Medical School, Germany

## Abstract

Electrophysiological oscillations in different frequency bands co-occur with perceptual, motor and cognitive processes but their function and respective contributions to these processes need further investigations. Here, we recorded MEG signals and seek for percept related modulations of alpha, beta and gamma band activity during a perceptual form/motion integration task. Participants reported their bound or unbound perception of ambiguously moving displays that could either be seen as a whole square-like shape moving along a Lissajou's figure (bound percept) or as pairs of bars oscillating independently along cardinal axes (unbound percept). We found that beta (15–25 Hz), but not gamma (55–85 Hz) oscillations, index perceptual states at the individual and group level. The gamma band activity found in the occipital lobe, although significantly higher during visual stimulation than during base line, is similar in all perceptual states. Similarly, decreased alpha activity during visual stimulation is not different for the different percepts. Trial-by-trial classification of perceptual reports based on beta band oscillations was significant in most observers, further supporting the view that modulation of beta power reliably index perceptual integration of form/motion stimuli, even at the individual level.

## Introduction

Electroencephalographic and magneto-encephalographic recordings on the human scalp reveal synchronized activity of large neuronal ensembles [1], [2]. A prominent feature of EEG-MEG activity is the characterization of oscillations in particular range of frequencies that correlates with different cognitive states [1], [3]. Similar oscillatory activity is found in the local field potential (LFP) in animal models [4], [5] or during intracranial recordings in epileptic patients [6], [7], suggesting it represents a genuine activity related to information processing that reflects specific neuronal network architecture [1], [8], [9]. The functional roles and relationships to perceptive, decisional, motor and cognitive processes of these oscillations however remain an open issue [3], [10], [11]. Amongst the family of cortical oscillations, alpha, gamma and beta band activities prompted a number of studies, owing to their co-occurrence with perceptual, attentional, decisional and motor processes. Gamma oscillatory activity (35–100 Hz) is frequently observed in a variety of studies and protocols [10], [12], [13] in relation with the binding of object's features processed in different brain regions, either across or within visual areas. Consequently, several authors proposed that gamma oscillations serve to facilitate the communication between neurons responding to distinct object's characteristics, setting-up synchronized neuronal ensembles able to encode the unified percept of a single object in a flexible way [5]. The functional role of gamma oscillations in perceptual binding is however still debated. Gamma oscillations do not reliably or exclusively index perceptual binding; studies targeted on binding processes sometimes failed to report reliable gamma activity or find decreased synchrony [14], [15], thus raising doubts on its functional role [14], [16], [17]. Other studies reported that gamma activity correlates with micro eye-movements and micro-saccade rate [18], [19], raising the possibility that gamma activity is modulated or possibly induced by small eye-movements. On the other hand, recent studies [20] pointed out prominent activity in the beta band (15–25 Hz) whose role is however debated [21]. Strong relationships between beta activity and motor processes have long been observed [22], beta rhythm being associated with preparation and inhibitory control in the motor system [23]. Recent studies also uncovered strong relationships with perceptual processing and beta oscillations. For instance, visual processing can be altered by trans-cranial magnetic stimulation (TMS) in the beta range [24]. Beta activity was also observed during binocular rivalry and with bistable stimuli [20], [25] suggesting a role for beta activity in visual processing. Alpha band activity is one prominent cortical rhythm which is modulated in a variety of experimental paradigms [26], [27] although its functional role in visual perception remains little understood [28]. Recent studies found that the alpha rhythm is present during the maintenance of sensory representations over time [29] or found modulations of alpha power in relation with objecthood [30]. Overall, these studies consistently report oscillatory activity in the gamma, beta and alpha range that occur in conjunction with cognitive processes.

The conditions favoring the emergence of gamma and beta activity within artificial neural networks endowed with different dynamics [8] brought evidence that gamma activity is prominent in a context of local excitatory/inhibitory interactions with short conduction times, whereas beta activity emerges for longer conduction delays. As conduction delays mainly depend upon the length of axonal connections, the dependence of oscillatory frequency on conduction delays in these artificial networks indirectly suggests that gamma and beta oscillations reflect the architecture of cortical circuits generating these oscillations. For instance, beta oscillations would reflect more specifically long, inter-areal synchronization, which is also supported by the fact that beta oscillations occur more frequently in deep layers receiving feedback inputs from distant regions [3].

Despite numerous reports of oscillatory activity in different frequency ranges during cognitive task, establishing correlations between cognitive processes and cortical oscillations is difficult because one cognitive task rarely recruits a single cognitive process: perception, attention, memory, motor preparation and execution are often required and their effects are mixed, making it difficult to parse the respective contribution and specificity of the processes at work. In this study, we analyzed the MEG physiological correlates of visual form/motion integration using well controlled elementary moving stimuli (see below). By further decoupling the motor response from the stimulation period, by having attention and decision evenly distributed amongst different trials and by minimizing and balancing the memory load across conditions, we could identify a strong and reliable bilateral parietal beta activity that distinguishes different perceptual states at the individual level and, for a significant proportion of participants, could be used to classify observers' reports on a trial-by-trial basis.

**Figure 1:**
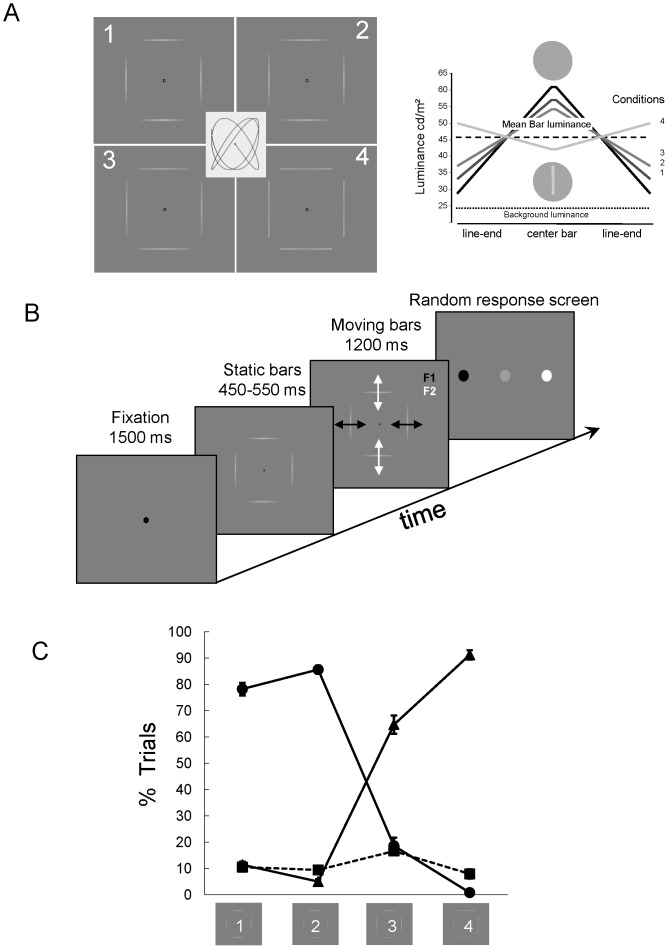
Stimuli and experimental protocol. A. Stimuli consisted of four disconnected bars arranged in a square shape whose contrast was modulated along the bars. When in motion these stimuli can be perceived as incoherent (unbound) pairs of bars oscillating along cardinal axes at different frequencies (2.3 and 3 Hz) or as a single (bound) square shape translating along a Lissajou's trajectory (inset). B. Time course of a trial: a trial started with the presentation of a fixation point for 1500 ms, followed by the presentation of the static bars for a variable duration (450–550 ms) after which the oscillatory motion began and lasted for 1200 ms. At the end of the motion stimulation, a screen appeared with response color codes scrambled on each trial, which implied remapping the motor response on each trial thus avoiding motor preparation and anticipation that could contaminate the data. C. Individual behavioral responses (dotted lines) and average responses (black line) for the 4 stimulus conditions. Bars with low contrast line-ends (conditions 1 and 2) are mostly seen as a bound shape, bars with high contrast line-ends (conditions 3 and 4) are mostly seen as unbound segments (Filled circles: percentage of the trials seen as bound; filled squares percentage of the trials seen as unbound; filled triangles: percentage of unclassified trials). Errors bars represent ±1 standard error.

We took advantage of the ‘aperture diamond’ stimulus [31] to probe perceptual integration. In this display, periodic oscillations of disconnected bars arranged in a square shape entail the perception of a solid square moving along a Lissajou's figure or the perception of disconnected bars oscillating independently. To test whether different bound/unbound percepts entail neural oscillations in different frequency bands, we relied on previous psychophysical studies showing that high contrast bar-ends favor motion segmentation while low contrast bar-ends favor motion integration [31]. Crucially, reliably eliciting bound and unbound percepts with these stimuli can be done by using subtle modulations of the distribution of contrast along the moving bars (Figure 1A), often unnoticed by observers, but that nevertheless entail drastic perceptual changes. It is out of the scope of the present study to detail the reasons why such small changes in local contrast flip the appearance of an otherwise identical stimulus. Let us just mention that surround suppression in V1 neurons is strongly modulated by contrast [33], [34] and is thought to exert a control on spatial pooling and motion integration [31], [32]–[35], suggesting that perceiving bound or unbound percepts is coupled to the modulation of V1 end-stopped responses.

In the following, we present the results of a MEG study where participants classified their perception of motion displays and analyze the power of oscillatory activity in the alpha, beta and gamma range in order to identify spectral fingerprints [36] of visual form/motion binding among these candidate markers.

## Material and Methods

### 1. Participants

Twelve naive right-handed volunteers with normal vision took part in the study (6 women and 6 men, mean age 29.3±9.1 years). All participants provided informed written consent and received a financial compensation for their participation. Two participants who misused the response buttons were excluded from the analyses. All procedures were approved by the local research ethics committee (Comité de Protection des Personnes Île-de-France VI, Paris, France).

### 2. Stimuli and procedure

The stimuli were presented via a mirror at the centre of a rear projection screen using a calibrated video projector (1024×768 pixels; refresh rate, 60 Hz) located outside the shielded recording room. The distance between subjects' eyes and the screen was 0.85 m. The subjects' head was inclined so as to favor the recordings of occipital MEG sensors [37]. The stimuli were composed of two pairs of horizontal and vertical bars (mean luminance 45.7 cd/m^2^; length 5.0 degrees of visual angle, dva thereafter; mean distance from the centre 3 dva) displayed on a grey background (mean luminance 23.6 cd/m^2^) and distributed around the central fixation point so as to form a square shape (6.8×6.8 dva) with invisible corners (Figure 1A). During a trial, the bars remained static for a short variable period (450–550 ms) and then moved for 1200 ms as follows: the horizontal bars oscillated in phase along a vertical axis at f1 = 2.3 Hz, while the vertical bars oscillated in phase along a horizontal axis at f2 = 3 Hz. This stimulus was expected to trigger the responses of direction selective cells at different harmonics of the bar motion frequencies, thus allowing the identification of neural populations responding to the vertical and horizontal motions. Motion amplitude was identical for the horizontal and vertical motion and equal to 1.2 dva.

As the perceptual binding of the component motions into a rigid moving shape is known to depend on the luminance ratio between the centre and line-ends of the bars [31], we designed four conditions, each characterized by a triangular distribution of luminance along the bars, as shown in Figure 1A. High-luminance line ends favor the perceptual segmentation into unbound oscillating bars, while lower-luminance line-ends favor their perceptual integration into a single moving shape. Preliminary behavioral experiments were conducted to choose luminance distributions yielding graded percepts: from strongly bound (condition 1, Video S1) to strongly unbound (condition 4, Video S2). Note that the mean luminance and motion distribution of the bars is identical in all conditions. These stimuli, when static, are hardly discriminated on the sole basis of their luminance distribution (Figure 1). In contrast, when the bars oscillate periodically along cardinal axes, the different stimuli elicited highly distinguishable perceptual states: a square with invisible corners moving rigidly along a Lissajou's trajectory - bound percept-, or pairs of bars moving independently along the vertical and horizontal axes - unbound percept. One observer who noticed the differences in luminance distribution was removed from the analyses. Although long observation of the present stimuli results in bistable percepts [38], [39], a procedure using physically different stimuli to favor different –bound/unbound- percepts was preferred for the following reasons: 1. In an fMRI study with similar stimuli, endogenous bistability or physical induction of perceptual fluctuations did not entail significantly different BOLD signals [38]. 2. Although bistable stimuli provide an elegant way of probing the mechanisms underlying different percepts with a single stimulus, the lack of a common base line temporally close to the stimulation, the intra- and inter-subject variability of the durations of each perceptual episode and the need to record perceptual switches on-line, which implies a motor response at any time, pose difficult challenges for the data analyses (e.g. contamination by the motor preparation and execution).

The time flow of experimental trials was as follows (Figure 1B): A fixation point was first presented on the screen for 1.5 second (t = −1.5 to t = 0). At t = 0, four static bars were displayed for a duration varying randomly between 450 and 550 milliseconds to avoid the confounding effect of stimulus and motion onset. This static phase was followed by 1.2 second of motion where both pairs of bars oscillated at fixed, although different, frequencies, along an axis orthogonal to their orientation. Finally, a response screen with three color-coded disks presented side by side was displayed to indicate which response was associated with each button of the keypad: black for a rigidly moving square –“bound percept”-, white for independent bar motions –“unbound percept”-, grey for an indecipherable percept or to signal an intrusive perceptual switch –“unclassified trials” thereafter. In order to minimize artifacts associated with motor preparation, the horizontal position of the three disks was randomly shuffled on each trial so that observers had to wait for the response screen before encoding and making their motor response. Each subject underwent 8 runs of 60 trials each (15 trials per condition) for a total of 120 trials per condition.

### 3. MEG recordings

Continuous magneto-encephalographic signals were collected at a sampling rate of 1250 Hz, using a whole-head MEG system with 151 axial gradiometers (CTF Systems, Port Coquitlam, British Columbia, Canada), and low-pass filtered on-line at 300 Hz. Before each run, head localization was measured with respect to the MEG sensors using marker coils that were placed at the cardinal points of the head (nasion, left and right ears). Eye movements were recorded with an ISCAN eye-tracking system (240 Hz sampling rate). We also recorded the signal of a photodiode that precisely detected when the bars appeared on the screen. This allowed us to correct for the time delays introduced by the video projector (∼24 ms) and to compute event-related magnetic fields (ERFs) precisely time-locked to the real stimulus onset.

### 4. Data analyses

Data were first pre-processed using both CTF and in-house software (http://cogimage.dsi.cnrs.fr/logiciels/). Trials contaminated by eye movements, blinks, or muscular artifacts were rejected off-line on visual inspection of ocular and MEG traces (as a result, 30% of the trials, evenly distributed amongst the 4 conditions, were discarded from further analyses: 27.6%, 30%, 29.5% and 31.4% for condition 1 to 4 respectively, corresponding to bound trials: 1582, unbound trials: 1436 trials; condition 1: 869 trials; condition 2: 840 trials; condition 3: 845 trials; condition 4: 823 trials). Time zero was set at the onset of motion using a photodiode signal. Global analyses were performed on all non-rejected trials independently of observers' percepts. Contrasts of MEG activity were also computed between trials classified as bound or unbound; excluding unclassified trials (8.9%). Analyses performed on averaged signals (SSVEF) time-locked to the stimulation have been presented elsewhere [16]. We here analyze the oscillating activity in the alpha, beta and gamma ranges to highlight variations induced by a bound versus an unbound percept.

#### 4.1 Sensors analysis

The analyses done on the MEG sensors involved a time–frequency wavelet transform applied on each trial in order to analyze the frequency components of the MEG signal induced by the stimulation. Time-frequency maps were computed for each MEG sensor using a family of complex Morlet wavelets (m = 10), resulting in an estimate of the signal power for each time sample and each frequency between 1 and 100 Hz with a resolution varying with the frequency (Wf = 0.235f in frequency and Wt = 3.74/f in time). Final time-frequency maps were obtained by further applying a base-2 log-transformation to the ratio of the signal power relative to the baseline, for each time and frequency sample (to correct for the 1/f distribution of the raw spectral power). As stated in the introduction, we focused the analysis on three frequency bands of interest: alpha, beta and gamma bands. As the peak value of those frequency bands depend on subjects [40], [41], we determined the bandwidth for each of these functionally defined oscillations by analyzing the pooled MEG signals including all the trials independently of the experimental conditions and percepts.

Alpha activities being predominant during the pre-stimulus baseline around occipital-parietal sensors (refs), we averaged the signal power during this epoch to characterize alpha power in our population. As a result, we found a sustained activity between 8 and 12 Hz, and used this alpha frequency band in subsequent analyses.

According to previous studies showing an occipito-parietal beta band deactivation during motion perception [42], we determined the relevant bandwidth of beta band oscillations by averaging baseline corrected activity on all occipito-parietal sensors for all the trials (i.e. independently of conditions and perception). In this way, we identified a sustained deactivation between 15 and 25 Hz, and thus used this range of interest in further analyses.

Finally, as previous studies identified gamma activity in occipital cortex using moving bars [43], [44], we identified the bandwidth of gamma activity in our population by averaging baseline corrected activity on occipital sensors. As a result, we found a sustained activity between 55 and 85 Hz which we took as the relevant gamma band in subsequent analyses.

In this way, the frequency bands of interest were determined independently of observers' reports using all the trials from all the experimental conditions. Unless otherwise mentioned, all the analyses on the sensors were conducted using these frequency bands.

#### 4.2 Statistical tests

To evaluate whether activity in these three frequency bands is modulated by perception during the course of a trial, we averaged the power within each band, resulting in a single time course per band of interest, sensor and subject. The significance of the differences in all performed contrasts was then established using a nonparametric cluster randomization test on a time window ranging from 0, corresponding to motion onset, to 1200 ms following the procedure proposed by Maris and Oostenveld [45], [46]. Signal samples whose T-value exceeded a first significance threshold (two-tail p-value <0.05) were clustered based on time and space adjacency, space adjacency being defined by the template matrix provided by FieldTrip (http://fieldtrip.fcdonders.nl/). Each cluster thus delineated was assigned a statistical value equal to the sum of the t-values over all the samples belonging to the cluster. To test whether this sum-of-t could be obtained by chance, the same clustering procedure was applied to the same data, but with the condition labels randomly reassigned. The clustering procedure was then applied on those randomized data, and the maximal sum-of-t was measured over the new clusters. By repeating the random assignment of the condition labels 1000 times, we could estimate the distribution of the maximal sum-of-t statistics under the null hypothesis. Because this method uses the maximum statistics, it intrinsically controls for multiple comparisons, and the null hypothesis can be rejected with a p-value of 0.05 when a cluster value of the original dataset is greater than 95% of the values obtained on randomized data. As this test was computed for three frequency bands, the p-threshold was decreased to 0.01.

#### 4.3 Trial-by-trial classification of perceptual states

To assess whether beta activity predicts individual observer's perceptual reports, we performed a trial-by-trial classification of the data using a modified Common Spatial Pattern method [47]. We conducted this analysis for each subject using an identical number of trials for each percept. To that aim, we first determined which perceptual report had least trials, and randomly picked-up an equivalent number of trials for each percept amongst the other trial sets. The number of trials used for the classification ranged from 65 to 173 (avg = 122, s.d. = 35) depending on the observers. The classification was computed from the raw signals filtered between 17-22 Hz (to take into account the frequency resolution of the complex Morlet wavelets, see above) over the sensors of interest derived from the clustering analysis (see above). For each subject, a fixed classifier was first obtained from a training set including 90% of the trials, chosen at random. The classification rates were then computed using a test set corresponding to the remaining 10% of the trials. This procedure was repeated 10 times. The classification rate of each observer was then taken as the mean of the 10 rates obtained in this way. To assess the significance of these classification rates, we derived a statistics from the whole data set, pooling bound and unbound trials, and computed a hundred times the classification rates of these trials with themselves. The resulting distribution of classification rates was then taken as a reference against which the subsequent classifications tests were compared. A classification rate was considered significant if, and only if, it was larger than 95% of the reference classification rates. Amongst the linear spatial filters of the fixed classifier, only the first 5 variables were used these for the classification tests.

#### 4.4 Source modeling

Sources of the MEG signals were estimated with the BrainStorm software (http://neuroimage.usc.edu/brainstorm) using a spherical head volume conductor and the cortical template “Colin27” of the Montreal Neurological Institute (MNI, http://www.bic.mni.mcgill.ca/). Co-registration of the anatomical template with the MEG coordinate system was achieved for each subject by aligning the positions of 3 reference coils with their corresponding anatomical landmarks (nasion and pre-auricular points). The MEG source imaging consisted of 10.000 elementary equivalent current dipole (ECD) sources distributed at each cortical node of the cortical tessellation and normal to the surface [48] [38]. We took into account the head position recorded at the beginning of each run to enhance the precision of the reconstruction. We used a minimum-L2-norm approach [49] [39] to obtain one time course for each subject, trial, and node of the cortical tessellation. As the duration of the motion onset asynchrony varied from trial to trial, signals were triggered to motion onset before averaging. For each of the 8 runs and for each subject, the responses were averaged across all trials and separately for bound and unbound trials. For each subject, the corresponding global responses were obtained with a weighted average across runs with respect to the numbers of trials of each category. This methodology takes head position recorded at the beginning of each run into account so as to enhance the precision of the reconstruction. For each significant difference found at the sensor level, we reconstructed the sources of the activity to localize the corresponding brain regions.

#### 4.5 Spectral Analysis on sources time courses

For each cortical source time courses, we estimated the power spectrum over two periods, one during the static display (baseline) and the other one during motion stimulation. The analysis of the stimulation period was conducted on 1200 ms of the moving stimulus and the baseline signal was estimated on a time window of same size, from −1800 ms to −600 ms before motion onset. The spectral analysis was performed using a Welch's periodogram [50] [40] associated with Hamming windows. This methodology allowed us to estimate the power for frequency-bands of interest for each trial and subject. A base-2 log-transformed ratio of the signal power relative to the baseline was taken as the measure of interest.

## Results

### 1. Behavioral results

The averaged distribution of observers' reports is presented in Figure 1C as a function of the 4 contrast conditions. As it can be seen, observers mostly perceived a single moving square when line-end contrast was low and perceived disconnected moving segments when line-end contrast was high. Overall, only few trials (8.9%) were unclassified, suggesting observers were confident in their choices and reliably classified their perceptual state over the duration of a trial. It is worth noting that whereas line-end luminance increases linearly across the first three conditions, observers' judgments show a discontinuity in the bound/unbound classification between condition 2 and 3, confirming that a small change in bar-end contrast entails drastic perceptual modifications. An ANOVA (3×4 factors) conducted on these data indicated a significant interaction between perception and condition (F = 114.79, p<0.05; η2 = 0.92). Additional analyses for each percept (1 factor, 4 conditions) showed a significant effect of the conditions on the response rate for the bound (F = 81.28, p<0.05; η2 = 0.891) and unbound (F = 93.58, p<0.05; η2 = 0.908) percept but not for unclassified percepts (F = 0.93, p = 0.44).

### 2. MEG results

Analyses of the steady state responses evoked by the oscillatory bar motions at the fundamental (2.3, 3 Hz), first harmonics (4.6, 6 Hz) and their intermodulation products have been presented elsewhere [16]. Briefly, increased power at the 10.6 Hz intermodulation product during bound states was found in frontal sensors, while the response power at the motion related frequencies of interest did not significantly differ as a function of perception on occipital or parietal sensors.

We here focus on the activities induced by the different perceptual states in three frequency bands (alpha: 8–12 Hz; beta: 15–25 Hz; gamma: 55–85 Hz), whose limits were first identified using all the trials (see above section Data analyses).

The analysis conducted using all the trials revealed modulation of gamma activity in occipital sensors (see figure 2 in [16]). In addition, decreased activity over the left motor cortex in the beta band (15–25 Hz) was observed, as expected considering that participants reported their percepts using their right hand and prepared to respond.

**Figure 2:**
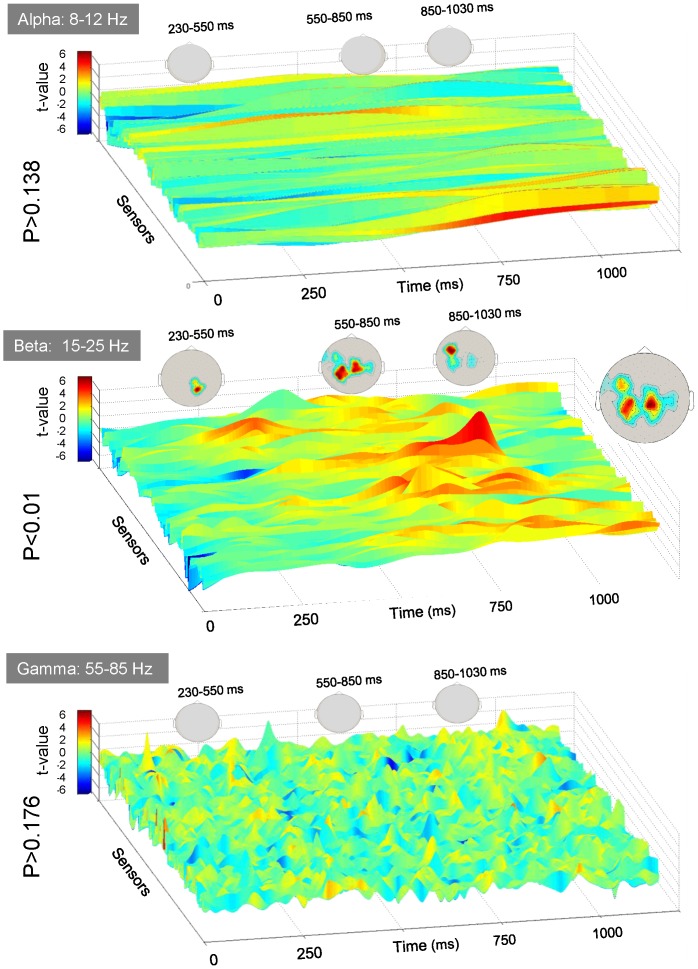
MEG results. A. Results of the spatio-temporal clustering analysis (see data analyses). In these plots, the t-values contrasting bound as compared to unbound percepts are shown for all the sensors as a function of time for alpha, beta and gamma oscillations. No significant differences were found for alpha (p>0.138) and gamma (p>0.176) frequencies. A significant cluster corresponding to more ample oscillations for bound as compared to unbound percept were found for the Beta band (p<0.01). Three topographies of sensors corresponding to three periods (230–550 ms, 550–750 ms and 750–1050 ms) are also shown. In these topographies, the color code denotes the time during which a sensor of a significant cluster was significantly different in bound as compared to unbound percepts.

The clustering analyses did not reveal any significant changes in the alpha and gamma power as a function of perceptual reports (alpha p>0.138; gamma p>0.176). In contrast, a sustained modulation of beta power (p<0.01) was found during motion stimulation. Figure 2 presents the t-values obtained when contrasting bound and unbound percepts for the alpha, beta and gamma frequency bands for all the MEG sensors as a function of time. As it can be seen, alpha and gamma oscillations are little modulated by perception and are not significantly different during bound and unbound percepts, in contrast with beta oscillations. Topographical maps of the significant cluster at different time intervals are also shown.

As it can be seen, the differences in beta power were overall sustained, although they smoothly developed during the time course of a trial over the scalp. Differential beta power emerged about 230 ms after motion onset over occipito-parietal sensors then developed toward centro-parietal around 550 ms, before reaching left frontal sensors about 750 ms after motion onset. Difference in beta power then dropped around 1100 ms after motion onset, slightly before the end of the visual stimulation.

**Figure 3:**
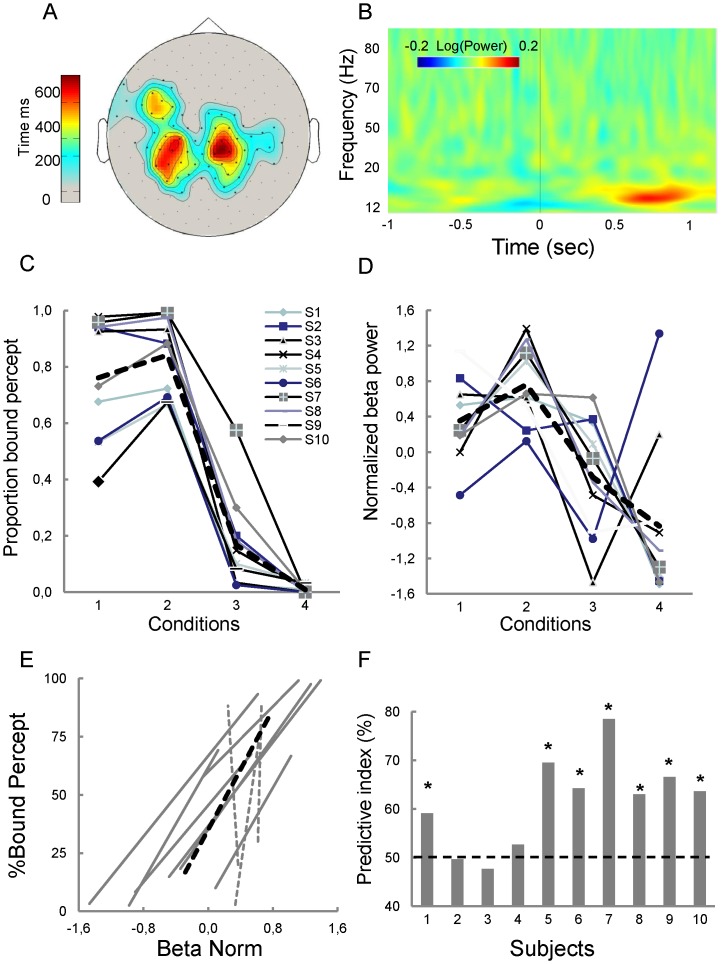
Comparisons between perceptual performance and beta power. A: Topography of the 63 sensors with significant difference between bound and unbound trials. In this plot, the color code denotes the time during which a sensor was significantly different in bound as compared to unbound percepts. B. Time frequency plots obtained by averaging sensors (n = 63) showing increased beta power for bound trials. C, D. Comparison of behavioral data (C) and normalized beta power (D) for each observer. The doted black line represents averaged data; light grey lines represent the data for each observer. E. behavioral responses for conditions 2 and 3 plotted as a function of normalized beta power. Normalized beta power increases with perceived motion coherence for all but 3 observers. F. Results of trial-by-trial classification (see method) using beta power averaged across frequencies (17–22 Hz) and time (100–1100 ms; see text for details). Significant classification (*, p<0.05) is obtained for 7 out of 10 observers.

The topography of all sensors belonging to the identified cluster showing significant beta modulations during motion stimulation is shown in figure 3A in which the color code denotes the time during which a sensor was significantly active. As it can be seen, activity is mostly localized on central-parietal sensors. To easily visualize the differences between alpha, beta and gamma oscillations, the time frequency plots encompassing all frequency bands of interest is shown on figure 3B. To more easily visualize the relationships between perceptual reports and beta activity, the later was normalized and plotted for each participant as a function of the experimental conditions. As it can be seen, the distribution of normalized beta power (figure 3D) closely resembles the distribution of reports of bound percepts (figure 3C). Plotting beta activity against the percentage of trials classified as bound (Figure 3E) for conditions 2 and 3 that are physically very similar (Figure 1A) further indicates that lower beta power is associated to fewer bound reports for the majority of observers (7/10), while higher beta power is associated to more frequent bound reports.

#### 2.1 Trial-by-trial classification

The striking similarity between perceptual reports (Figure 3C) and normalized beta power (Figure 3D) suggested that beta power is a reliable marker of perceptual states. To test further this eventuality, we sought to recover participants' reports on each trial on the basis of this sole activity. This classification (see material and methods) was computed using the sensors and time window revealed by the clustering analysis. As a result of this classification test, a significant proportion of the individual trials were correctly classified for 7 out of 10 subjects (Figure 3F). These results corroborate those of previous studies [20], [51] that relied on beta power modulation to classify perceptual reports of ambiguous or noisy stimuli. We here confirm and extend these previous findings to the perceptual form-motion integration processes involved by our stimulus design.

#### 2.2 Source reconstruction

**Figure 4:**
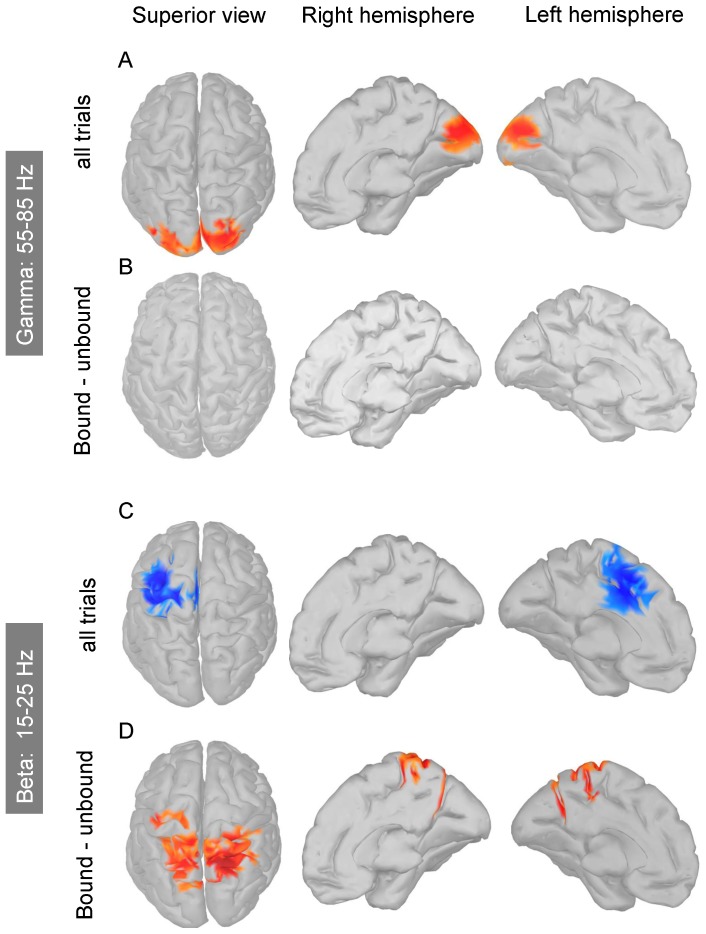
Reconstruction of the sources of gamma and beta band oscillations. Only clusters of contiguous sources (n>50) with power greater than 50% of maximum activity are shown. A. Sources of gamma power (range 55–85 Hz) computed using all the trials are mainly located within the occipital lobe. B. Power differences between bound and unbound trials fail to reveal sources in the gamma band. C. Sources of beta power (15–25 Hz) computed using all the trials show decreased beta power over the left motor cortex, as expected given that observers used their right hand to report their perception. D. Sources of beta power computed from the differences between bound and unbound trials are mostly distributed over central-parietal cortex.

Reconstructing the sources of both the gamma and beta power for all the trials and for the differences between bound and unbound trials refined the loose localization of these activities on the scalp. As a result, shown in figure 4, we found that the sources of the gamma activity were mainly confined to the occipital lobe while the sources of the decreased beta power, found when all the trials are collapsed, lie over the left motor cortex. In contrast, the sources of the difference in beta power between trials seen as bound and unbound are mostly confined to the central-parietal cortex, although some sources around the motor cortex also show increased beta power related to the bound/unbound reports. Overall, the distributions of these sources confirm the conclusions drawn from the sensor activity.

## Discussion

In this study, we analyzed the magneto-encephalographic activity recorded during subjective reports of stimuli either seen as a bound square moving along a Lissajou's trajectory or as unbound bars oscillating independently along cardinal axes. We found strong gamma activity over the occipital lobe during visual stimulation but this activity was not modulated as a function of the perceptual reports. As expected, alpha band activity decreased after stimulus onset, but this modulation was similar for all perceptual states. In contrast, beta band power, which overall decreased over the motor cortex in the left hemisphere during stimulation compared to baseline, as expected given observers used their right hand to indicate their choice, was more important during bound as compared to unbound percepts. This differential beta activity spread over the scalp during a trial, emerging ∼250 ms after motion onset over occipital-parietal sensors before shifting after 550 ms toward central-parietal sensors, followed by left frontal sensors after 850 ms, and disappearing ∼150 ms before a trial ended. Reconstructing the sources of the differential beta activity during the motion stimulation mainly revealed a bilateral central-parietal region, although some sources overlap the region of global decreased beta activity found over the left motor cortex when including all the trials in the analysis. In addition, the correlation between beta power and perception at the individual level allowed significant trial-by-trial classification of the perceptual reports, indicating that beta band activity fluctuates as a function of perceptual state in most participants.

Several accounts of the observed differential beta oscillations are to be considered. They could simply reflect the physical contrast of each stimulus condition and not merely perceptual integration. Alternately, as is often the case, the task may have engaged attention, decision, motor preparation, implicit trial timing or memory, which could be involved to varying degrees depending on perception. We discuss these eventualities in the following.

### 1. Physical contrast or perceptual motion integration?

In this study, small modulations of line-end bar contrast entailed clear and salient modifications in perception, despite the overall averaged contrast being kept the same across conditions. One may thus be concerned that the modulation of beta power described here is caused by these physical modulations alone. Disentangling perceptual states from physical modifications, for instance by comparing bound and unbound reports for the same condition, is however uneasy because the number of trials reported as bound or unbound is not evenly distributed. For example, the conditions 2 and 3, although physically very similar (12.5% and 19.3% line-end contrast respectively), are mostly classified as bound and unbound, respectively, leaving few trials to dissociate the effects of physical changes and perceptual reports, such that statistical analyses are irrelevant. However, if line-end contrast *per se* accounted for the change in Beta power, one would expect that Beta power increases -or decreases- in proportion to increasing contrast, which is not the case. In addition, if contrast was effectively driving the cortical responses, the signals directly evoked by the oscillatory motions should also be modulated by contrast. We previously reported that the responses at the fundamental and 1^st^ harmonics of the oscillatory motions were not statistically different for the different conditions [16], disproving the idea that differences in physical contrast entailed significantly different evoked responses. Moreover, it is unclear why physical contrast would modulate oscillatory power specifically in the beta band and not in other frequency bands, gamma band in particular [43]. The drastic influence of small contrast differences on perceptual integration calls for non-linear effects able to shift a percept of unbound segmented moving bars into that of a bound integrated moving object. Although caused by changes in line-end contrast, such non linear effects presumably reflect cortical processing of the incoming inputs, possibly related to released end-stopped inhibition at low line-end contrast [33], [52], which in turn facilitates the neuronal communication underlying motion integration across time and space, while enhanced end-stopping for high-contrast line-ends may prevent the integration of contour and motion into a whole. Although this may indicate that beta oscillations are related to modulation of end-stopping inhibition, further studies are needed to address this issue.

### 2. Perceptual integration or other cognitive processes?

Several studies comparing EEG and MEG activity elicited by attended and unattended stimuli report attention dependent oscillations in the alpha and/or gamma range, or in the steady-state visually evoked responses power, SSVEP [12], [44], [53]. Can a different distribution of attention during trials seen as bound or unbound account for the present results? Although participants were to attend similarly to all conditions to perform the task, one cannot exclude that allocation of attention differed during bound, as compared to unbound, percepts. According to previous results, a percept dependent shift in attention should elicit modulations of alpha, gamma power and/or SSVEP. The analyses done to test this eventuality indicate that gamma power, although strong and reliable during visual stimulation, was independent of perceptual reports. The lack of significant differences in alpha power for the different percepts similarly argues against an effect of attention. If oscillations in these frequency bands reliably index an increased attentional load toward object-like stimuli, the lack of significant differences in alpha and gamma activities suggests that allocation of attention did not consistently differ as a function of perception in this study. Finally, previous studies using temporally modulated stimuli [54] reported attentionnal power modulations at the harmonics of the periodic stimulations. If attention was to account for the differences reported here, one would also expect similar differences in the activity evoked by the oscillatory bar motions, which was not the case [16].

Another possibility is that beta activity reflects the development of decisional processes, at stake when asked to classify stimuli. For instance, reaching a decision might be more difficult in condition 2 and 3 that are physically more similar than conditions 1 and 4 that are more different. More difficult decisions for uncertain stimuli could result in differences in beta power, as has been reported [51] in a motion detection task that required integrating motion evidence over several seconds to cross a motion threshold. In this situation, gamma power correlates with motion strength, while increased beta power appears to reflect the accumulation of evidence leading to a decision. In the present study with supra-threshold stimuli, beta band oscillations do not seem to reflect decision uncertainty, as beta power is different for the more uncertain conditions 2 and 3 and for the more certain conditions 1 and 4 (Figure 3E). In addition, observers could use an “unclassified” response button whenever they found it hard to classify their percept. Those few “unclassified” trials were evenly distributed across conditions (Figure 1) and discarded from the analyses, such that more uncertain or difficult decisions may not have contributed much to the beta modulation reported here. Finally, a decision has to be made for bound as well as unbound percepts. It is unclear why neural activity would differ much for bound and unbound decisions, except if it is related to the perceptual content rather than the decision process itself. Altogether, these considerations weaken the possibility that enhanced beta power during bound reports reflects decisional processes independently of perceptual processing.

Alternately, observers could have made their decision quickly and maintained their choice until the response screen appeared, which necessitates keeping their choice in memory until a motor response can be made. In line with previous reports relating cortical oscillations and memory [55], [56], beta activity could index this process, a view compatible with the observation that the difference in beta power reaches a maximum around 800 ms after motion onset. Although we cannot refute this interpretation at this stage, it is unclear why memory load would significantly differ for bound and unbound decisions (except if the perceptual content rather than the decision itself is kept in memory). Similarly, motor preparation and motor response are unlikely to explain the present findings because motor preparation is needed whatever the perceptual report and can only be produced after the randomized stimulus-response mapping screen was displayed, after the end of the visual stimulation.

Overall, because the cognitive processes needed to perform the task are balanced across conditions and perceptual reports, we think that the paradigm used in this study is well suited to recruiting and isolating the neural correlates of perceptual form/motion integration and limits the possibility that uncontrolled cognitive processes elicited unbalanced activity for bound and unbound reports.

### 3. Perceptual and beta power dynamics

If beta power index perceptual integration, the observation that the difference in beta power between bound and unbound trials emerges about 230 ms after motion onset is puzzling, as perceptual integration is expected to emerge soon after motion onset. However, psychophysical studies of the dynamics of motion integration with drifting plaids or with aperture stimuli similar to those used herein indicate that motion integration develops slowly (100–300 ms), as evidenced by dynamic shifts in perceived direction [57] or coherence [58], or directly probed through the recordings of direction selective MT cells [59]. In this study, the beta power for bound and unbound percept diverges after a delay comparable to perceptual dynamics and stabilizes after motion integration is completed and perception reaches a stable state. In this view, beta oscillations could help sustaining the perceptual outcome of a bound shape until a response is given.

The present results add to recent findings of consistent relationships between cortical oscillations and perceptual and cognitive processes [2], [3], [53], [60]. A consensual view is that oscillations in different frequency bands are spectral fingerprints [36] whose characteristics are constrained by the architecture of the neural connectivity within and between regions [36], [61]. In this regard, cortical oscillations provide insights into both the processes involved in a perceptual task and the neural substratum of the underlying dynamic ensemble [3]. Gamma oscillations (35–100 Hz) are often considered to index local sensory processes within a cortical region, owing to short range excitatory and inhibitory interactions elicited by an incoming sensory input (e.g. in layer 4), a view supported by modeling [8] as well as electrophysiological and neuroimaging studies [4], [5], [62]. In contrast, beta oscillations (15–30 Hz) are thought to reflect long-range interactions facilitating information transfer between cortical regions [3], [11], and appear to mainly originate from feedback projections in superficial and deep cortical layers [63], [64]. It has further been suggested that beta oscillations develop and maintain over time so as to sustain a “status quo” [21] during which perception is stabilized, providing matter to decision and action. The present results fit well with this scheme. Sustained gamma oscillations found over the occipital lobe when including all the trials possibly index the local interactions recruited to encode each oscillating bar independently. In line with previous reports, larger beta activity could reflect increased neuronal communication across neuronal populations coding for the different bars in different cortical sites during the encoding of a moving bound shape, which engages mechanisms of motion integration, contour completion, surface filling-in, depth ordering as well as the computation of border ownership [39], [65]–[68] [34], [51]–[54]. In line with the observation that neurons in parietal regions receive convergent projections from visual areas responding to global motion [69], [70] or to shape [71], enhanced beta oscillations could facilitate the integration of oscillating bars into a moving shape in central-parietal regions. Analyzing long-range coherence, synchronization and functional connectivity between sources showing beta modulations and sources involved in processing the stimulus (as revealed by the SSVEF analysis and alpha and gamma power in all conditions) could further reveal how beta oscillations emerge from the visual stimulus processing and contribute to perceptual decision.

## Conclusion

Modulations of beta power over central-parietal regions provide a marker of perceptual integration, allowing significant trial-by-trial classification of observers' reports. This is not the case for gamma and alpha oscillations whose power is independent of perceptual states. This pattern of results fits well in a general framework in which beta oscillations would facilitate the neuronal communication underlying the perceptual integration of oscillating disparate elements into a moving whole, while gamma activity involving short-distance interactions would subtend the encoding of incoming sensory inputs.

## Supporting Information

Video S1 **Video of the moving stimulus condition 1, with low-contrast line-endings, mostly perceived as a bound square moving along a Lissajou's trajectory.**
(MPG)

Video S2 **Video of the moving stimulus condition 4, with high-contrast line-endings, mostly perceived as unbound segment pairs undergoing vertical and horizontal oscillatory motion.**
(MPG)
